# Can antioxidants protect against chemotherapy in a rat spermatogonial stem cell line?

**DOI:** 10.1530/RAF-21-0042

**Published:** 2021-11-16

**Authors:** Caroline M Allen, Federica Lopes, Rod T Mitchell, Norah Spears

**Affiliations:** 1 Biomedical Sciences, University of Edinburgh, Edinburgh, UK; 2 MRC Centre for Reproductive Health, University of Edinburgh, Edinburgh, UK

**Keywords:** antioxidants, chemotherapy, fertility preservation, spermatogonial stem cells

## Abstract

Boys administered chemotherapy to treat cancer are at risk of damage to their healthy testicular tissue, which can lead to infertility in adulthood. Researchers are therefore investigating treatments to protect the testis during cancer treatment. Here, cells originating from rat testicles were cultured for 4 days and exposed to chemotherapy drugs with or without antioxidants for the final 2 days. Antioxidants can reduce cellular damage by inactivating toxic compounds. Here, antioxidants such as melatonin or *n*-acetylcysteine were tested against chemotherapy agents cisplatin, doxorubicin, or vincristine. Cultures were repeated four times, with cell survival measured at the end of culture. The antioxidants were not damaging and partially protected against cisplatin, although not doxorubicin. Surprisingly, *n*-acetylcysteine enhanced vincristine-induced damage. The results suggest that using antioxidants to protect the testis could have either beneficial or harmful effects when given alongside different chemotherapy drugs: this is important, considering that patients are often treated with multiple drugs.

Children undergoing chemotherapy to treat childhood cancers are at risk of infertility in adulthood due to drug cytotoxicity (Allen *et al.* 2018). The development of fertility preservation strategies is therefore vital, particularly for prepubertal boys for whom there is a lack of clinically available options (Goossens *et al.* 2020). Cytoprotective agents included in cancer treatment regimens could act to preserve fertility by protecting the reproductive tissues against chemotherapy-induced damage. Some studies have reported a role for reactive oxygen species (ROS) in chemotherapy-induced testicular toxicity (Allen *et al.* 2018). The potential for antioxidants to protect the testis has been investigated in a number of clinical and animal model studies; however, its use is controversial due to the lack of evidence of effectiveness (Yasueda *et al.* 2016).

Here, the ability of antioxidants such as melatonin or *n*-acetylcysteine (NAC) to protect against chemotherapy-induced cytotoxicity was investigated in rat GC-6spg cells, which represent spermatogonial stem cells (SSCs), based on the expression of expected markers including promyelocytic leukemia zinc finger (PLZF) (van Pelt *et al.* 2002, Carlomagno *et al.* 2010) (Fig. 1A). While this immortalised cell line displays some differences in morphology and/or physiology compared to SSCs *in vivo*, these cells are still capable of migration into the SSC niche upon injection into testes, providing further evidence of SSC characteristics (van Pelt *et al.* 2002). Chemotherapy agents investigated include cisplatin, doxorubicin, and vincristine, all commonly used to treat childhood cancers. To date, ROS production has been linked to cisplatin and doxorubicin-induced toxicity but not in relation to vincristine (Conklin 2004). The cell line had been stabilised at passage 53 and was cultured here at passage 62 as described (van Pelt *et al.* 2002), at density 50,000 cells/cm^2^ until 80% confluent (48 h). Cells were exposed to either serial dilutions of antioxidant, cytotoxic chemotherapy concentration, co-treatment or vehicle for a further 48 h. Cytotoxicity was determined by MTT (3-(4,5-dimethylthiazol-2-yl)-2,5-diphenyltetrazolium bromide) assay to measure cell viability based on metabolic activity; cells were exposed to 0.5 mg/mL of MTT for 3 h, formazan product solubilised in 1:1 isopropanol:DMSO and solution absorbance analysed at 560 nm. Absorbance of treated cells was compared to control to give percentage of cell viability per control, with background accounted for blank wells.

**Figure 1:**
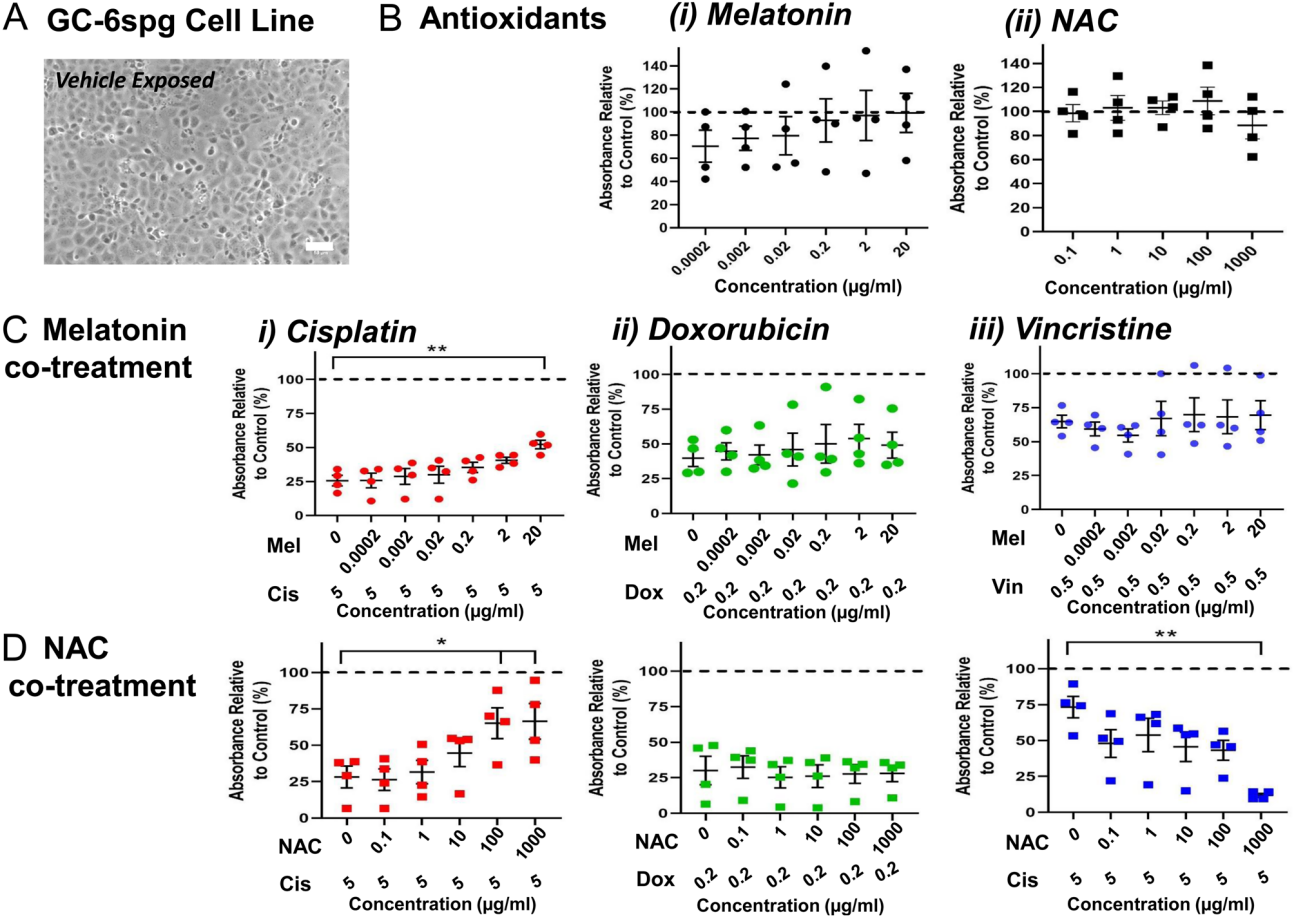
Effects of antioxidant co-treatment on chemotherapy-induced cytotoxicity in GC-6spg cell line. Both melatonin and NAC protected against cisplatin-induced cytotoxicity, whereas NAC exacerbated vincristine-induced cytotoxicity. (A) Representative image of GC-6spg cell line vehicle exposed at end of the culture, scale bar 50 µm. (B) Impact of (i) melatonin (0.0002–20 µg/mL) or (ii) NAC treatment (0.1–1000 µg/mL) on cell viability. (C) Effects of melatonin co-treatment on (i) cisplatin- (5 µg/mL), (ii) doxorubicin- (0.2 µg/mL) or (iii) vincristine (0.5 µg/mL)-induced cytotoxicity. (D) Effects of NAC co-treatment on (i) cisplatin- (5 µg/mL), (ii) doxorubicin- (0.2 µg/mL) or (iii) vincristine (0.5 µg/mL)-induced cytotoxicity. (B) One sample T-test, comparing the viability of treated cells to vehicle control deemed 100% viable (dashed line). (Ci) One-way ANOVA and Dunnett’s multiple comparisons* post-hoc* test or (Cii-iii/Di-iii) Kruskal–Wallis and Dunn’s multiple comparison (data required non-parametric statistical analyses) to compare viabilities of co-treated cells to chemotherapy-exposed cells. *P  < 0.05, **P  < 0.01. Data shown as mean ± s.e.m. where data points are representative of average viabilities from a minimum of three replicate wells from each of four separate culture runs, *n*  = 4.

Antioxidants did not affect cell viability in comparison to vehicle controls (considered as 100% viable): shown as dotted line in Fig. 1B (*P* > 0.05). Co-treatment with melatonin or NAC partially protected against cisplatin damage at the highest antioxidant doses (*P*  < 0.05, Fig. 1C/Di); cisplatin treatment decreased viability to ~25%, whereas melatonin (20 µg/mL) co-treated cells were 52 ± 3% viable (*P*  < 0.01; Fig. 1Ci) and NAC co-treated cells 65 ± 11% and 67 ± 12% viable at 100 and 1000 µg/mL, respectively (*P*  < 0.05 for both; Fig. 1Di). No protective effects were found following antioxidant co-treatment of doxorubicin-exposed cells (*P* > 0.05, Fig. 1C/Dii). This is in alignment with previous data, where treatment with antioxidants vitamin C, curcumin, amifostine, or carnitine did not protect against doxorubicin-induced loss of viability in GC-6spg or Sertoli cell lines (Tremblay & Delbes 2018). Here, melatonin co-treatment did not protect against vincristine-induced cytotoxicity (Fig. 1Ciii), whereas with NAC, not only was there a lack of protection but also it significantly enhanced cytotoxic effects of vincristine (Fig. 1Diii): cell viabilities were reduced to 12 ± 1% upon 1000 µg/mL co-treatment compared to vincristine where cells were 73 ± 7% viable (*P*  < 0.01). Additional research is required to determine the underlying action of antioxidants upon interaction with chemotherapy agents, whether due to effects on proliferation and/or apoptosis.

These findings suggest a potential role for melatonin/NAC in protecting against cisplatin-induced damage at concentrations that exceed doses used clinically. Further research is needed to determine whether antioxidant regimens with lower concentrations for a longer period could be as effective; this would be required to demonstrate clinical relevance. Furthermore, findings show that different antioxidants can have positive or negative effects on chemotherapy-induced cytotoxicity, with NAC-enhancing vincristine cytotoxicity. This is important when considering clinical relevance, since treatments almost always include multiple drugs that may react differently to potential protectants. In addition, further studies are required to determine whether antioxidants interfere with cytotoxic action upon cancer.

## Declaration of interest

Norah Spears is Co-Editor-in-Chief of *Reproduction and Fertility* and was therefore not involved in the review or editorial process associated with this paper, of which she is a co-author. Federica Lopes is an Early Career Editor of *Reproduction and Fertility* and was therefore not involved in the review or editorial process associated with this paper, of which she is a co-author.

## Funding

This work was funded by Career Development PhD Scholarship and Children with Cancer UK grant #15-198. R T M’s is supported by a UKRI Future Leaders Fellowship (MR/S017151/1) in the MRC Centre for Reproductive Health funded by MRC Centre Grant MR/N022556/1. C M A was supported by Career Development PhD Scholarship in Biomedical Sciences funded by the Biomedical Sciences ZJU project.

## Author contribution statement

C M A: conceived and participated in the experimental design of the study, led experiments, analysed data, prepared figure and wrote the manuscript; F L: participated in the experimental design of the study and commented on earlier versions of the manuscript; R T M: participated in the experimental design of the study and commented on earlier versions of the manuscript; N S conceived, designed and coordinated the study and helped draft the manuscript. All authors read and approved the final version of the manuscript.
